# Awareness, knowledge, attitude and practice of adverse drug reaction reporting among health workers and patients in selected primary healthcare centres in Ibadan, southwestern Nigeria

**DOI:** 10.1186/s12913-019-4775-9

**Published:** 2019-12-03

**Authors:** Rasaq Adisa, Tomilayo I. Omitogun

**Affiliations:** Department of Clinical Pharmacy and Pharmacy Administration, Faculty of Pharmacy, University of Ibadan, Ibadan, Nigeria

**Keywords:** Adverse drug reaction reporting, Awareness, knowledge, attitude, and practice, Primary healthcare worker, Patients

## Abstract

**Background:**

Higher incidence of adverse drug reactions (ADRs) is a global health problem requiring attention of all stakeholders regardless of the practice settings. This study therefore aimed to evaluate awareness, knowledge, attitude and practice of ADR reporting among health workers and patients in 10 primary healthcare centres (PHCs) in Ibadan, southwestern Nigeria.

**Methods:**

Questionnaire-guided cross-sectional survey among 80 health workers and 360 patients enrolled from the selected PHCs between October and December 2018. The semi-structured questionnaires generally comprised open-ended and closed-ended questions to explore general knowledge and awareness of ADRs and pharmacovigilance, while other question-items evaluated attitude towards ADR reporting and ADR reporting practice. Overall percent score in the knowledge and attitude domains for the health workers was developed into binary categories of > 80 versus ≤80% for “adequate” and “inadequate” knowledge, as well as “positive” and “negative” attitude, respectively. Data were summarised using descriptive statistics, while Chi-square test was used to evaluate categorical variables at *p* < 0.05.

**Results:**

Overall, 58(72.5%) health workers had heard of pharmacovigilance**,** but only 3(5.2%) correctly understood the pharmacovigilance concept. Twelve (15.0%) showed adequate knowledge of ADRs, while 37(46.2%) demonstrated positive attitude towards ADR reporting. Thirty (37.5%) health workers had come across ADR reporting form, while 79(98.8%) expressed willingness to report all ADRs encountered. Of the patients, 31(8.6%) had heard of pharmacovigilance, 143(39.7%) correctly cited ADR definition, while 67(18.6%) reported the previously experienced ADRs. Informing healthcare professional (38; 38.8%) was the most common measure taken by patients when they experienced reaction(s). Nurses significantly had adequate knowledge of ADRs (*p* < 0.001) compared to other cadres.

**Conclusions:**

Health workers in the selected PHCs were largely aware of pharmacovigilance but show low level of knowledge about ADRs and pharmacovigilance concept, with moderately positive attitude towards ADR reporting. Patients on the other hand demonstrate low level of awareness of pharmacovigilance and ADR reporting, with less than one-fifth who reported the previously experienced ADRs. This perhaps underscores a need for regular mandatory education and training on ADRs/pharmacovigilance concept among the PHC health workers, while continuous public enlightenment and awareness campaign on spontaneous reporting of ADRs is advocated in order to enhance reporting rate.

## Background

Higher incidence of adverse drug reactions (ADRs) is a global health problem requiring attention of all stakeholders regardless of the practice settings [1, 2]. Adverse drug reactions have been reported as a significant cause of morbidity and mortality across all age groups, with appreciable number of hospital admissions, as well as substantial financial burden on the society and the healthcare systems [2, 3]**.** Spontaneous ADR reporting system is a global phenomenon, and the cornerstone of pharmacovigilance activities [4]. Healthcare professionals in any capacity therefore play a crucial role in pharmacovigilance system, and as such they require considerable knowledge and expertise in the field of medication safety, especially for early recognition, detection, management and reporting of ADRs [5]. However, studies have indicated that a large proportion of ADRs are not reported by healthcare professionals, especially in developing countries [6–9] due to a number of factors including lack of awareness and knowledge of pharmacovigilance and ADR reporting [5–7, 10]. It is estimated that only 6–10% of all ADRs are reported [11, 12]. Thus, underreporting has been a major obstacle to spontaneous reporting of ADRs, and this poses a great challenge to pharmacovigilance activities [4, 7] as well as negatively impact public health [7, 13]. Healthcare professionals are therefore expected to consider ADR reporting as their professional obligation, since an effective system of ADR reporting is important to improve patient care and safety [14] and in turn improving overall health [15, 16]. Similarly, patients play a vital role in spontaneous reporting system, as only the patient really feels the adverse effect of the drug [17]. The success or failure of any spontaneous reporting system depends on the active involvement of reporters [18]. Thus, direct patient participation in ADR reporting will increase efficiency of the pharmacovigilance system, as well as bridge the gap of underreporting on the part of healthcare professionals [19, 20].

Previous studies on pharmacovigilance and ADR reporting in Nigeria and many other developing countries [6, 8–10, 14], focused mostly on healthcare practitioners in secondary and tertiary care settings with less attention paid to the primary healthcare centres (PHCs), which are the closest to members of the community in most developing countries, as well as being a recognized centre for public health programmes [21]. This study is therefore designed to evaluate awareness, knowledge, attitude and practice of ADR reporting among health workers and patients attending 10 selected PHCs in Ibadan, southwestern Nigeria.

## Methods

### Study site

Ibadan, the capital city of Oyo state in the southwest geopolitical zone of Nigeria comprised 11 Local Government Areas (LGAs) with 234 PHCs widely distributed across the nooks and crannies of the LGAs with different population size and coverage. Of the 11 LGAs, five LGAs were used for this study including Ibadan North (urban), Ibadan North East (urban), Ibadan South West (urban), Lagelu (semi-urban) and Akinyele (semi-urban) which were classified into urban and semi-urban LGAs based on their strategic and geographical locations within the city. There are about 114 PHCs spread across the five LGAs with different level of patients’ patronage depending on their location within the LGA. Each LGA has a model PHC located either within or in close proximity to the LGA headquarters which is usually the prototype. A total of between 120 and 200 adult patients above 18 years were estimated to patronize the model PHC on a regular basis per month, while between 6 and 10 health workers were on full-time employment in each PHC. The 10 PHCs selected for this study comprised the model PHC for each LGA and another PHC chosen based on patients’ patronage. The PHCs were Idi-Ogungun and Agbowo from Ibadan North LGA with a coverage of 4 km and 6 km, respectively; Iwo Road and Alafara from Ibadan North East LGA with a coverage of 4 km and 9 km, respectively; Bolumole (3 km) and Foko (4 km) from Ibadan South West LGA; Alegongo (5.6 km) and Olorunda (10 km) from Lagelu LGA; Moniya and Sasa PHCs from Akinyele LGA with a coverage of 2 km and 6 km, respectively.

### Study design

A questionnaire-guided cross-sectional survey among health workers and patients attending the selected PHCs in Ibadan, southwestern Nigeria between October and December 2018.

### Study population

All the PHC workers on full-time employment as healthcare practitioner as well as adult patients attending the selected PHCs for medical care.

### Sampling techniques

The 11 LGAs in Ibadan were stratified into urban and semi-urban LGAs, of this, five LGAs consisting of three urban and two semi-urban LGAs were selected using a systematic random sampling of every other LGA. Subsequently, 10 PHCs were purposively selected from the five LGAs comprising the model PHC for each LGA and another PHC chosen based on relatively high patients’ patronage. In each PHC, total sampling of health workers on daily duty and consecutive sampling of patients receiving care in the PHC facility were done.

### Inclusion and exclusion criteria

Full-time health workers involved in day-to-day treatment and care of patients at each PHC, as well as adult patients attending the PHC for health-related complaints and who gave consent for participation were enrolled. Workers in the PHCs who were solely employed for administrative duties, health workers on in-service or pre-qualification training as well as patients who declined participation were excluded.

### Sample size determination

Sample size for the study was determined using Raosoft® sample size calculator [22]. In each selected PHC, average of 40 adult patients regularly attend the facility per week, given an estimated population of 3200 patients from the 10 PHCs for the 8 weeks study period (i.e. 40 × 10 × 8 = 3200). Also, an average of eight health workers were on full-time employment in each PHC facility, given an estimated population of 80 health workers from the 10 PHCs (i.e. 10 × 8 = 80). Thus, computing the estimated population of the adult patients and health workers into the sample size calculator, at 95% confidence level and 5% margin of error gave a sample size of approximately 345 patients and 67 health workers. However, the inclusion of a 10% non-response rate gave a target sample size of approximately 380 for patients and 74 (rounded off to 80) for health workers, to guide participants’ enrolment.

### Data collection instrument

The questionnaires for the study were developed by the investigators following extensive review of relevant studies [7–9, 11, 19], as well as utilizing previous practice experience. The semi-structured questionnaire for health workers consisted of four sections that explored demographic information, awareness and understanding of pharmacovigilance concept, as well as general knowledge about ADRs. Also, health workers’ perceived attitude towards ADR reporting was evaluated using nine item-statements with 5-points Likert scale response option, while ADR reporting practice among the health workers was also captured**.** The 5-points ordinal scale ranged from strongly agree (5) to strongly disagree (1) for positive statements (1–6), and a reversed ranked score for the negative statements (7–9). The questionnaire for the patients comprised three sections with question-items that captured socio-demographic characteristics, knowledge and awareness of pharmacovigilance and ADR reporting as well as ADR reporting practice (see Additional file 1).

### Pretest and validation of the instrument

The questionnaires were assessed for content validity by two academic scholars who were expertise in the field of pharmacovigilance and ADRs. This is to ascertain the comprehensiveness of the item-statements in the questionnaires *vis-à-vis* the study objectives, as well as ensuring that there are no ambiguous questions or statements. Subsequently, pretest/face validity of the questionnaires was done among five PHC health workers and 15 patients recruited from one of the excluded PHCs, so as to ascertain the ease of comprehension of the question-items by would-be participants, as well as the appropriateness of the recruitment procedure. Feedback from the pretest and validity assessments led to minor modifications in the questionnaires such as the re-designing of some statements with a Yes/No response option as a ranked variable so as to ensure clarification of opinion.

### Data collection procedure

Health workers were courteously approached when visited in their respective PHCs, largely during the scheduled duty shift period for individual worker and when there are less patients’ workload. Writing material, specifically pen, was made available for those who may not have it handy at the time of filling the questionnaire. This approach was consistently applied throughout the period of the study, pointedly to ensure optimal cooperation and sustained interest of the health workers. Patients on the other hand, were enrolled for participation while waiting for their turn of treatment by the health workers. The informed consent form in the approved protocol, which captured the consent information including objectives and procedure of the study, risks and benefits of participation, as well as expected duration of involvement among others, was read and explained to individual participant prior to questionnaire administration. Subsequently, verbal informed consent was obtained from every participant to signify their intention for participation. The questionnaire for the health workers which took between 20 and 25 min to complete was self-administered by the workers and subsequently cross-checked for completeness before the disengagement of any participant. The interviewed-administered questionnaire to the patients which took about 25 to 30 min to complete, was done by the principal investigator. The patient’s questionnaire and the informed consent form were translated into Yoruba, the local language for ease of understanding. Back-translation to English was later done to ensure response consistency. Participants’ anonymity and confidentiality of responses were assured, while they were informed that participation in the study is entirely voluntary.

#### Data analysis

Data obtained were sorted, coded and entered into Microsoft Excel spreadsheet for ease of data management, and subsequently the computed data was exported into SPSS version 20.0 for analysis. Descriptive statistics including frequency and percentage was used to summarize the data. The sum total of score for the health workers in the knowledge and attitude domains was converted into percentage score, and subsequently developed into a binary category. In the knowledge domain, a total correct score > 80% (i.e. > 10 out of the 13 maximum obtainable score) was assigned as “adequate” general knowledge of ADRs and reporting, while a total score ≤ 80% was classified as “inadequate” knowledge. In the attitude domain, a total ranked score > 80% (i.e. > 36 out of the 45 maximum points) signified “positive” attitude towards ADR reporting, while a total ranked score ≤ 80% was classified as “negative” attitude. The cut-off for the overall percent score in the knowledge and attitude domains was adapted from Bloom’s cut-off point criteria, as well as other related studies [23, 24]. Pearson Chi-square or Fischer’s Exact test as appropriate was used to evaluate association between healthcare workers’ demographic variables and the overall knowledge and attitude scores. Association between patients’ socio-demographic characteristics and ADR knowledge, as well as pharmacovigilance awareness and reporting of experienced ADRs was also done using Pearson Chi-square or Fischer’s Exact test. The level of statistical significance was set at *p* < 0.05.

## Results

### Demographic characteristics of health workers

All the 80 health workers enrolled within the study period responded to the questionnaire, given a response rate of 100%. Seventy-four (92.5%) were females and 6 (7.5%) were males. The professional cadres of health workers who participated in the study included community health extension worker (30; 37.5%), community health officer (24; 30.0%), nurse (20; 25.0%), health assistant (4; 5.0%) and physician (2; 2.5%). Majority, 53 (66.3%) had practice experience within ≤1–5 years and 13 (13.3%) had > 5 years’ experience.

### Knowledge and awareness of pharmacovigilance and ADRs among the health workers

Fifty-eight (72.5%) health workers had heard of pharmacovigilance, of this, most (21; 36.2%) became aware through other healthcare professionals. Only 3 (5.2%) understood the comprehensive definitions of pharmacovigilance. Forty-eight (60.0%) believed that ADRs can only be experienced by a patient using orthodox medicines. Overall, 12 (15.0%) of the health workers had total score > 80% suggesting “adequate” knowledge of ADRs (see Table 1).

**Table 1:** Awareness and knowledge of pharmacovigilance and adverse drug reactions among the health workers

Question/variable	Frequency	Percent
Have you heard of pharmacovigilance? (*n* = 80)		
Yes	58	72.5
No	22	27.5
If yes, source of awareness of pharmacovigilance (*n* = 58)		
From other health professionals	21	36.2
Through seminar and training	18	31.0
Medical/Nursing school	9	15.5
Advertisement	9	15.5
Internet	1	1.7
Wha What is your understanding of pharmacovigilance? (*n* = 58)		
The detection, assessment, and prevention of adverse effects and other drug-related problems	24	41.4
The science and activities involved in reporting adverse drug reactions	14	24.1
The process by which adverse drug reactions are monitored in a hospital	10	17.2
A practice focused on medication and patient safety	7	12.1
All of the above	3	5.2
13-item knowledge questions in adverse drug reactions (*n* = 80)	Response, n (%)	
	YES	NO
1.An adverse drug reaction is a side effect that is commonly experienced when patient is using a drug	53 (66.3)	27 (33.7)^a^
2. An adverse drug reaction is an unintended effect of the drug during its administration	65 (81.2)^a^	15 (18.8)
3. An adverse drug reaction is a predicted and expected reaction to a drug	33 (41.2)	47 (58.8)^a^
4. An adverse drug reaction is the same as a side effect	22 (27.5)	58 (72.5)^a^
5. An adverse reaction can only be experienced by a patient using orthodox medicines	48 (60.0)	32 (40.0)^a^
6. An adverse drug reaction can be experienced by a patient using herbal/traditional medicines	45 (56.2)^a^	35 (43.8)
7. All adverse drug reactions are known before drug gets into the market for use	21 (26.2)	59 (73.8)^a^
8. All adverse drug reactions experienced by a patient taking a drug should be reported and documented	76 (95.0)^a^	4 (5.0)
9. Only intolerable reactions to a drug should be reported	25 (31.2)	55 (68.8)^a^
10. Adverse drug reaction may not be documented if the patient was appropriately counselled on such reaction	25 (31.2)	55 (68.8)^a^
11. The best method of addressing adverse effect is to use or recommend another drug	51 (63.8)	29 (36.2)^a^
12. There is no need to report an adverse drug reaction that is already documented in drug literature insert	24(30.0)	56(70.0)^a^
13. Reporting and documentation of adverse drug reaction is important	76(95.0)^a^	4(5.0)
Overall cut-off for knowledge score (%)	Frequency (%)	Remark
Score > 80	12 (15.0)	Adequate
Score ≤ 80	68 (85.0)	Inadequate

^a^Correct answer, maximum obtainable score = 13, percent knowledge score = Individual score divided by 13 multiply by 100

### Health worker’ perceived attitude towards ADR reporting

Seventy-nine (98.8%) of the health workers expressed willingness to report all the ADRs encountered, 74 (92.5%) believed that ADR reporting is part of their professional responsibilities as a healthcare professional, while all the health workers (80; 100%) agreed that pharmacovigilance concept should be included as a component of training for PHC workers. Overall, 37 (46.2%) had a total score > 80% indicating “positive” attitude towards ADR reporting (see Table 2).

**Table 2:** Evaluation of health workers’ perceived attitude towards adverse drug reaction reporting

Attitudinal statements (*n* = 80)	SA & A	U	D & SD
1.I would report all adverse drug reactions I encountered	79 (98.8)	0 (0.0)	1 (1.3)
2. ADR reporting is part of my responsibilities as a healthcare professional	74 (92.5)	5 (6.3)	1 (1.3)
3. Training of healthcare professionals can aid adverse drug reaction reporting	77 (96.2)	0 (0.0)	3 (3.8)
4. I would be more likely to identify and report important adverse drug reactions if I received training on pharmacovigilance	73 (91.3)	7 (8.8)	0 (0.0)
5. Reporting adverse drug reaction is part of my professional obligation	71 (88.8)	7 (8.8)	2 (2.5)
6. Pharmacovigilance concept should be included in the training of healthcare workers	80 (100.0)	0 (0.0)	0 (0.0)
7. I would likely report only life-threatening/severe adverse drug reactions	44 (55.0)	3 (3.8)	33 (41.3)
8. I would likely report only previously unknown adverse drug reactions	23 (28.8)	7 (8.8)	50 (62.5)
9. I do not think that tolerable, mild adverse drug reactions should be reported	25 (31.3)	8 (10.0)	47 (58.8)
Overall cut-off for attitudinal score (%)		Frequency (%)	Remark
Score > 80		37 (46.2)	Positive
Score ≤ 80		43 (53.8)	Negative

Items 1 to 6 are positive statements with rank score as strongly agree (SA) = 5, agree (A) = 4, undecided (U) = 3, disagree (D) = 2, strongly disagree (SD) =1, while items 7 to 9 are negative statements with reverse scoring as: SA =1, A = 2, U = 3, D = 4, SD = 5. Maximum obtainable score = 45 points. Percentage score obtained = individual score divided by 45, multiply by 100

### Adverse drug reaction reporting practice among the health workers

Forty-seven (58.8%) health workers had come across ADRs in their respective PHC. Measure(s) taken by those who had encountered ADRs were cited in combination to include referral to a secondary healthcare facility (50; 54.3%), as well as treating the symptoms with another drug (30; 32.6%) Table 3. Challenges preventing the health workers from reporting ADRs included unavailability of the reporting form (40; 37.4%), insufficient clinical knowledge (35; 32.7%), lack of experience in filling the ADR reporting form (10; 9.4%), non-threatening nature of ADRs (10; 9.4%), complicated nature of ADR reporting (7; 6.5%), fear of liability (4; 3.7%) and lack of time to report the ADRs (1; 0.9%). Thirty (37.5%) of the health workers had seen the ADR reporting form (Yellow form), but 17 (21.3%) had the reporting form in their facility. A total of 11 (13.8%) health workers had previous training in pharmacovigilance and ADR reporting, but 73 (91.3%) expressed willingness to received training in pharmacovigilance and/or ADR reporting. Acquisition of more knowledge (56; 70.0%) was largely mentioned as reason for the interest in pharmacovigilance/ADR training (see Table 3).

**Table 3:** Adverse drug reaction reporting practice among the health workers

Variable	Frequency (%)
Ever encounter adverse drug reaction (ADR) at health facility (*n* = 80)	
Yes	47 (58.8)
No	33 (41.2)
Measure(s) taken in the case of encountered ADRs (*n* = 92)^a^	
Refer to a secondary health facility	50 (54.3)
Treat symptoms with another drug	30 (32.6)
Provide counselling to patients	7 (7.6)
Stop the use of the drug	5 (5.4)
Ever come across ADR reporting form (*n* = 80)	
Yes	30 (37.5)
No	50 (62.5)
Is ADR reporting form available in your facility? (*n* = 80)	
Yes	17 (21.3)
No	63 (78.8)
Barrier(s) in obtaining the ADR reporting form (*n* = 17)	
No known source of obtaining the form	9 (5.3)
No proper distribution of form	4 (2.4)
Not the duty of primary healthcare	4 (2.4)
Who should report ADRs? (*n* = 91)^a^	
Any healthcare worker	50 (71.4)
Patients	26 (28.6)
Only senior healthcare worker	15 (16.5)
Ever received training on pharmacovigilance and/or ADR reporting? (*n* = 80)	
Yes	11 (13,8)
No	69 (86.2)
Opinion on willingness to receive training (*n* = 80)	
Yes	73 (91.2)
No	7 (8.8)
Self-rating score on interest to undergo training in pharmacovigilance (*n* = 73)	
< 5	0 (0.0)
5–7	8 (11.0)
8–10	65 (89.0)
Reasons for the interest to undergo pharmacovigilance training (*n* = 80)	
Acquire more knowledge on ADRs and reporting	56 (70.0)
Know more about the detection, assessment and prevention of ADRs	15 (18.7)
Patient care and safety	4 (5.0)
Easy access to ADR reports and early treatment of ADRs	3 (3.8)
More knowledge on ADRs, especially on how to fill the form	2 (2.5)
Regulatory bodies responsible for monitoring ADRs (*n* = 138)^a^	
NAFDAC	46 (33.3)
PCN	35 (25.4)
FMOH	30 (21.7)
WHO	27 (19.6)

*NAFDAC* National Agency for Food and Drug Administration and Control, *PCN* Pharmacists Council of Nigeria, *WHO* World Health Organisation, *FMOH* Federal Ministry of Health, *ADR* Adverse Drug Reaction

^a^Multiple response

Table 4 shows the association between health workers’ demographic characteristics and pharmacovigilance awareness, as well as overall ADR knowledge and attitude towards ADR reporting. Nurses (66.7%) constituted those who significantly showed adequate general knowledge of ADRs compared to other professional cadres including physician (16.7%), community health extension workers (16.7%), community health officers (0.0%) and health assistant (0.0%) [Fisher’s Exact Test (FST) = 21.44, *p* < 0.001]. Also, health workers with ≤1–5 years practice experience in the PHC (68.8%) significantly demonstrated positive attitude towards ADR reporting compared to those who had > 5 years’ experience (31.2%) [Pearson Chi-square (X^2^) = 5.24, *p* = 0.02] see Table 4. There was a significant association among health workers with or without prior pharmacovigilance training and general knowledge of ADRs (X^2^ = 4.57, *p* = 0.03), as well as pharmacovigilance awareness (FET = 4.84, *p* = 0.03). Health workers with no prior training (66.7%) in pharmacovigilance constituted those who significantly showed adequate general knowledge of ADRs compared to those with training (33.3%).

**Table 4:** Association between health workers’ demographic characteristics and pharmacovigilance awareness, adverse drug reaction knowledge and attitude towards adverse drug reaction reporting

Variables	ADR knowledge		Attitude towards ADR reporting		PV Awareness	
	Adequate (Score > 80%)	Inadequate (Score ≤ 80%)	Positive (Score > 80%)	Negative (Score ≤ 80%)	Yes	No
Age (year)						
18–40	9 (75.0)	49 (72.1)	26 (70,3)	32 (74.4)	39 (67.2)	19 (86.4)
> 40–60	3 (25.0)	19 (27.9)	11 (29.7)	11 (25.6)	19 (32.8)	3 (13.6)
	FET = 0.044; *p* = 1.000		X^2^ = 0.172; *p* = 0.679		X^2^ = 2.925; *p* 0.087	
Gender						
Male	1 (8.3)	5 (7.4)	2 (5.4)	4 (9.3)	3 (5.2)	3 (13.6)
Female	11 (91.7)	63 (92.6)	35 (94.6)	39 (90.7)	55 (94.8)	19 (86.4)
	FET = 0.014; *p* = 1.000		FET = 0.435; *p* = 0.681		FET = 1.647; *p* = 0.338	
Marital status						
Single	6 (50.0)	15 (22.1)	6 (16.2)	15 (34.9)	17 (29.3)	4 (18.2)
Married	6 (50.0)	53 (77.9)	31 (83.8)	28 (65.1)	41 (70.7)	18 (81.8)
	X^2^ = 4.113; *p* = 0.043^*^		X^2^ = 3.580; *p* = 0.058		X^2^ = 1.020; *p* = 0.312	
Educational qualification						
Primary	0 (0.0)	1 (1,5)	0 (0.0)	1 (2.3)	0 (0.0)	1 (4.5)
Secondary	0 (0.0)	2 (2.9)	0 (0.0)	2 (4.7)	1 (1.7)	1 (4.5)
Tertiary	12 (100.0)	65 (95.6)	37 (100.0)	40 (93.0)	57 (98.3)	20 (90.9)
	FET = 1.051; *p* = 1.000		FET = 6.506; *p* = 0.021^*^		FET = 7.847; *p* = 0.017^*^	
Professional cadre						
Nurse	8 (66.7)	12 (17.6)	7 (18.9)	13 (30.2)	19 (32.8)	1 (4.5)
CHO	0 (0.0)	24 (36.3)	15 (40.5)	9 (20.9)	15 (25.9)	9 (40.9)
CHEW	2 (16.7)	28 (41.2)	12 (32.4)	18 (41.9)	20 (35.4)	10 (45.5)
Health assistant	0 (0.0)	4 (5,9)	1 (2.7)	3 (7.0)	2 (3.4)	2 (9.1)
Physician	2 (16.7)	0 (0.0)	2 (5.4)	0 (0.0)	2 (3.4)	0 (0.0)
	FET =21.435; *p* < 0.001^*^		FET = 6.555; *p* = 0.136		FET = 9.139; *p* = 0.039^*^	
Years of experience in PHC						
≤ 1–5	10 (90.9)	43 (78.2)	22 (68.8)	31 (91.2)	35 (74.5)	18 (94.7)
> 5	1 (9.1)	12 (21.8)	10 (31.2)	3 (8.8)	12 (25.5)	1 (5.3)
	FET = 0.939; *p* = 0.678		X^2^ = 5.242; *p* = 0.022^*^		FET = 3.514; *p* = 0.088	
Previous training in PV						
Yes	4 (33.3)	7 (10.3)	7 (18.9)	4 (9.3)	11 (19.0)	0 (0.0)
No	8 (66.7)	61 (89.7)	30 (81.1)	39 (90.7)	47 (81.0)	22 (100.0)
	X^2^ = 4.565; *p* = 0.033		X^2^ = 1.551; *p* = 0.213		FET = 4.838; *p* = 0.030^*^	

*FET* Fischer’s Exact Test, *X*^*2*^ Pearson Chi-square, ^*^significant difference with Fischer’s Exact or Pearson Chi-square test, *PV* Pharmacovigilance, *ADR* Adverse drug reaction, *CHO* Community Health Officer, *CHEW* Community Health Extension Worker, PHC Primary Healthcare Centre. Level of statistical significance *p* < 0.05

### Socio-demographic characteristics of patients

A total of 360 out of the 380 eligible patients approached for participation in the study gave consent and completed the study, thus, a response rate of 94.7% was obtained among patients. Females constituted 311 (86.4%) and males were 49 (13.6%). Majority, 306 (85.0%) were within the age range of 18–40 years, while 54 (15.0%) were above 40 years. Most (187; 51.9%) of the patients were self-employed, 96 (26.7%) were civil/public servant and 77 (21.4%) had no active employment. Patients who had no formal education or at most a primary education were 32 (8.9%), secondary (148; 41.1%) and post-secondary/tertiary education were 180 (50.0%).

### Knowledge and awareness of pharmacovigilance and ADR reporting among patients

Thirty-one (8.6%) patients had heard of the term “pharmacovigilance”, mostly (9; 29.0%) through the social media platform. A total of 143 (39.7%) patients correctly understood what can be regarded as an ADR. Seventeen (4.7%) had prior knowledge of the short message service (SMS) alert short code for reporting experienced ADRs, with majority (13; 76.5%) who got aware of the code through advertisement and online source (see Table 5).

**Table 5:** Patients’ knowledge and awareness of pharmacovigilance and adverse drug reaction reporting

Variable	Frequency (%)
Ever heard of pharmacovigilance (*n* = 360)	
Yes	31 (8.6)
No	329 (91.4)
If yes, the source of pharmacovigilance awareness (*n* = 31)	
Social media platform	9 (29.0)
Television	5 (16.1)
Newspaper	5 (16.1)
Radio	3 (9.7)
Previous educational experience	3 (9.7)
Internet	3 (9.7)
A close healthcare professional	3 (9.7)
What is your understanding of an adverse drug reaction? (*n* = 360)	
Any effect from a medication one is taken	168 (46.7)
Unexpected reaction after taking a medication	143 (39.7)^a^
Expected reaction	16 (4.4)
I do not know	33 (9.2)
A serious adverse drug reaction means: (*n* = 363)^b^	
A reaction that may lead to hospitalisation	163 (44.9)
A reaction that is life-threatening	133 (36.6)
A reaction that requires another drug treatment	52 (14.3)
A reaction that resolves on its own	14 (3.9)
I do not know	1 (0.3)
Ever heard of SMS short code for reporting adverse drug reactions (*n* = 360)	
Yes	17 (4.7)
No	343 (95.3)
If yes, source of awareness of the SMS alert short code (*n* = 17)	
Advertisement and online sources	13 (76.5)
Newspaper articles	3 (17.6)
Friends	1 (5.9)

*n* number, *SMS* Short message service

^a^Correct answer, ^b^multiple response

### Adverse drug reaction reporting practice among patients

Eighty-nine (24.7%) of the patients had experienced one form of ADR or the other. Informing a healthcare provider (38; 38.8%) and stoppage of the drug (21; 21.4%) topped the list of actions taken by patients who had experienced ADRs. Direct reporting of experienced ADRs (197; 54.2%) to healthcare professional was the most preferred method of ADR reporting among patients. Sixty-seven (18.6%) of the patients reported the previously experienced ADRs. Suggested reasons for non-report of experienced ADRs included ignorance of the importance of ADR reporting (111; 30.8%), as well as unserious nature of the ADRs (105; 29.2%) see Table 6.

**Table 6:** Adverse drug reaction reporting practice among the patients

Variable	Frequency	Percent
Have you ever experienced an adverse drug reaction? (*n* = 360)		
Yes	89	24.7
No	271	75.3
Action taken in the case of adverse effect/reaction (*n* = 98)^**^		
Informed a healthcare professional	38	38.8
Stopped the drug(s)	21	21.4
Did nothing because the reaction was tolerable	17	17.3
Did nothing because the reaction resolved on its own	11	11.2
Used another drug to treat symptoms of reaction	7	7.1
Switched to herbal/traditional medicines	3	3.1
Switched to another drug	1	1.1
Sources of obtaining information about adverse drug reactions (*n* = 531)^**^		
Drug leaflet	172	32.4
Pharmacist	134	25.2
Physician	82	15.4
Internet	81	15.3
Nurse	58	10.9
Relative/Friend	4	0.7
Suggested reasons why patients do not report experienced adverse drug reactions (*n* = 360)		
Do not know the importance of reporting adverse drug reactions	111	30.8
Adverse reaction may not be very serious	105	29.2
Do not know how to report such reactions	86	23.9
Not sure if adverse reaction is related to the medication(s) used	53	14.7
Adverse effect/reactions resolved on its own	5	1.7
Preferred methods of adverse drug reaction reporting (*n* = 363)^**^		
Reporting directly to healthcare professional	197	54.2
Phone call or text message	108	29.7
Online application designed for adverse drug reaction reporting	26	7.2
Filling a reporting form	20	5,5
Filling an online reporting form	12	3.3

*n* number, *ADR* Adverse drug reaction ^**^ multiple response

Table 7 shows the association between patients’ socio-demographic characteristics and pharmacovigilance awareness, as well as ADR knowledge and reporting of previously experienced ADRs. Patients who had post-secondary/tertiary education were those with better pharmacovigilance awareness (90.3%), ADR knowledge (58.0%) and ADR reporting practice (56.7%) compared to those with lower educational qualification (*p* > 0.05). Patients who were within the age of 18–40 years (71.6%) constituted those who largely reported the experienced ADRs compared to those above 40 years (28.4%) [X^2^ = 11.52, *p* = 0.001] see Table 7.

**Table 7:** Association of patients’ socio-characteristics with pharmacovigilance awareness, knowledge of adverse drug reaction and reporting of experienced reactions

Variables	Knowledge of ADR definition		PV Awareness		Reporting of experienced ADRs	
	Adequate	Inadequate	Yes	No	Yes	No
Age (year)						
18–40	118 (82.5)	188 (86.6)	25 (80.6)	281 (85.4)	48 (71.6)	258 (88.1)
41 and above	25 (17.5)	29 (13.4)	6 (19.4)	48 (14.6)	19 (28.4)	35 (11.9)
	X^2^ = 1.147; *p* = 0.284		X^2^ = 0.505; *p* = 0.478		X^2^ = 11.521; *p* = 0.001^*^	
Gender						
Male	19 (13.3)	30 (13.8)	4 (12.9)	45 (13.7)	8 (11.9)	41 (14.0)
Female	124 (86.7)	187(86.2)	27 (87.1)	284 (86.3)	59 (88.1)	252 (86.0)
	X^2^ = 0.021; *p* = 0.884		FET = 0.014; *p* = 1.000		X^2^ = 0.195; *p* = 0.658	
Marital status						
Single	55 (38.5)	60 (27.6)	10 (32.3)	105 (31.9)	13 (19.4)	102 (34.8)
Married	84 (58.7)	152 (70.0)	20 (64.5)	216 (65.7)	52 (77.6)	184 (62.8)
Widowed/Divorced	4 (2.8)	5 (2.3)	1 (3.2)	8 (2.4)	2 (3.0)	7 (2.4)
	FET = 4.973; *p* = 0.083		FET = 0.478; *p* = 0.801		FET = 6.343; *p* = 0.037^*^	
Occupation						
Civil/public servant	41 (28.7)	55 (25.3)	13 (41.9)	83 (25.2)	21 (31.3)	75 (25.6)
Self-employed	71 (49.7)	116 (53.5)	11 (35.5)	176 (53.5)	30 (44.8)	157 (53.6)
No active employment	31 (21.7)	46 (21.2)	7 (22.6)	70 (21.3)	16 (23.9)	61 (20.8)
	X^2^ = 0.607; *p* = 0.738		X^2^ = 4.757; *p* = 0.093		X^2^ = 1.728; *p* = 0.421	
Educational qualification						
No formal education/Primary	6 (4.2)	26 (12.0)	1 (3.2)	31 (9.4)	4 (6.0)	28 (9.6)
Secondary	54 (37.8)	94 (43.3)	2 (6.5)	146 (44.4)	25 (37.3)	123 (42.0)
Post-secondary/Tertiary	83 (58.0)	97 (44.5)	28 (90.3)	152 (46.2)	39 (56.7)	142 (48.5)
	X^2^ = 9.594; *p* = 0.008^*^		FET = 23.619; *p* < 0.001^*^		X^2^ = 1.820; *p* = 0.402	

*FET* Fischer’s Exact Test, *X*^*2*^ Pearson Chi-square, ^*^significant difference with Fischer’s Exact or Pearson Chi-square test, *PV* Pharmacovigilance, *ADR* Adverse drug reaction. Level of statistical significance *p* < 0.05

## Discussion

In this study, close to three-quarters of the health workers were aware of pharmacovigilance, but approximately 5% correctly understand the comprehensive definitions of pharmacovigilance, while about one-sixth show adequate knowledge of ADRs. The proportion of health workers with inadequate level of ADR knowledge in our study is much lower than those reported in previous studies [25–27]. The disparity in ADR knowledge among participants from different studies may in part be linked to the possible variations in the criteria for determining the cut-off point, as well as differences in the study settings. However, the low level of knowledge of ADRs and pharmacovigilance among the PHC health workers may perhaps be connected to the fact that a larger percentage of health personnel at the PHCs in Nigeria can be regarded as paramedical group of healthcare professionals who may not be statutorily mandated to possess a bachelor degree in their health-related disciplines, thus, there may be a greater likelihood that pharmacovigilance or subject related to ADRs might not have been included in their training curriculum. Worthy of note to mention is the fact that the two physicians who participated in this study were among those who had adequate knowledge of ADRs as well as pharmacovigilance awareness. Though, overall, nurses were found to have significantly adequate general knowledge of ADRs compared to other professional cadres. The low level of awareness of pharmacovigilance and inadequate knowledge of ADR reporting have been widely reported among healthcare professionals generally [26–29]. It is also noted that about one-seventh of the health workers had prior pharmacovigilance or ADR-related training. Lack of training opportunities in pharmacovigilance has been reported in previous studies in Nigeria [8, 30], consequently most healthcare professionals had not received training on ADR detection and reporting [8, 31, 32]^.^ However, our study reveals that those without prior training in pharmacovigilance significantly constituted those with adequate general knowledge of ADRs and pharmacovigilance awareness compared to those with training. Even though the reason for this perceived disparity may not be clearly ascertained from our study. However, it may be necessary to consider the fact that for any formal training geared towards transfer and acquisition of knowledge such as pharmacovigilance or ADR related training, the appropriateness of the content, the quality of resource materials as well as the suitability of training delivery mode to facilitate learning and the expertise of resource person constitute important factors that may largely dictate the extent to which participants can grasp and internalize the content. Thus, in conducting a pharmacovigilance or ADR–related training, it may be essential to ensure that all the aforementioned attributes are featured in the training concept/idea in order to enhance knowledge acquisition by participants. Interestingly, over 90% of the health workers signify interest to undergo training in pharmacovigilance and/or ADR reporting, with approximately 90% who gave a self-rated score of between 8 and 10 as evidence to show for their level of interest in the training. Reasons cited largely include acquisition of more knowledge, especially in ADR detection, assessment, reporting and prevention. The overwhelming interest of health workers to receive training in pharmacovigilance needs to be further pursued by relevant authorities, perhaps through initiation of a mandatory continuing education programme on pharmacovigilance and/or ADR reporting for the PHC health workers across the country. Providing continuous educational activities, as well as cooperation among relevant stakeholders to disseminate information has been identified as effective intervention tool in improving spontaneous reporting [16, 33–35]. Of note is the fact that all the health workers supported the incorporation of pharmacovigilance concept into their training curriculum.

Overall, less than half of the health workers demonstrate positive attitude towards ADR reporting. Even though, healthcare providers play a significant role in ensuring a robust pharmacovigilance system, the rate of spontaneous reporting of ADRs by healthcare professionals in many countries is extremely low (6–10%), which may in part be due to the fact that spontaneous ADR reporting is not a mandatory requirement in most countries [11, 12].. However, in our study, perusing the response of health workers to specific attitude statement, it is observed that a substantial proportion expressed an encouraging positive attitude towards ADR reporting, although, the perceived positive attitude may not be a direct reflection of what may happen in clinical practice. Nevertheless, if this promising positive attitude towards ADR reporting among the PHC health workers can be further reinforced and pursued, perhaps through consistent and continuous training of the health workers, there may be improvement in ADR reporting rate. More especially considering the fact that the PHC health workers are the healthcare professionals who are closest to the people of the grassroot [21] who may largely be exposed or constitute a more vulnerable population to the eventual consequence(s) of ADRs. Overall, the health workers who are younger in the practice (≤1–5 years) significantly demonstrate positive attitude towards ADR reporting compared to those with greater than 5 years practice experience. Incorporation of ADRs and pharmacovigilance themes into the educational institutions and curricula where health disciplines are taught may therefore be an important use of formal educational channels, to actively incorporate ADR/pharmacovigilance concept into the future healthcare professional career, with the potential of strongly influencing perception and attitude towards ADR reporting [36]^.^

In this study, nearly 60% of the health workers had encountered an ADR case(s) in their practice site, and most of those who had come across ADRs took measures such as referral of the case to a secondary healthcare facility, while some engaged in treating the ADR symptom(s) with another drug. These actions taken by the health workers can be regarded as a mix of good and poor practice with respect to ADRs. In Nigeria and perhaps some other resource-poor countries, healthcare practitioners at the PHCs are usually expected to handle minor ailments, as well as involve in follow-up of patients with uncomplicated chronic disease conditions, thus, referral of ADR case(s) to a secondary healthcare facility may constitute a good practice by the health workers that needs to be encouraged. However, recommending another drug to treat an ADR symptom(s) may not be considered as a rational or good practice, since the act may possibly results into further negative consequences, especially when the cause of the initial reaction has not been fully explored.

In this study, a larger proportion of health workers expressed willingness in reporting all the ADRs encountered, however, a number of challenges preventing ADR reporting were cited, which includes unavailability of reporting form in the PHC, as well as insufficient clinical knowledge and experience in filling the ADR reporting form among others. Nonavailability of ADR reporting form and non-existence of a formal reporting system are consistent with previous studies [8, 27, 31, 37], thus, an issue that needs to be proactively addressed by concerned stakeholders, most especially the National Pharmacovigilance Centre (NPC) in the case of Nigeria. Ensuring availability and proper distribution of ADR form across the healthcare facilities from time to time, as well as embarking on continuous enlightenment and education of PHC health workers may be some of the measures to explore in order to ensure improvement in ADR reporting rate. Studies have indicated the availability of ADR reporting form (i.e. Yellow form), as well as a user-friendly reporting form as impetus for improving ADR reporting practice [35, 36].

Our study also reveals that less than 10% of the patients had heard of pharmacovigilance, while nearly 5% were aware of the short message service (SMS) alert short code for reporting ADRs but more than one-third display a good understanding of what can be regarded as an ADR. Previous studies in developed and developing countries have also reported low level of awareness of pharmacovigilance activities [6, 38]. Of note is the fact that most patients who heard of pharmacovigilance became aware through the social media platform, as well as news media, largely television and radio. News and social media platforms have been recognized as potential sources of gathering data on patients’ experience with medicines, though there are concerns about its effectiveness in supplementing routine pharmacovigilance activities [18, 19, 39]. Nevertheless, online sources and other contemporary technology, if properly utilized may be used to sensitize and create awareness about pharmacovigilance generally and ADR reporting in particular, with subsequent improvement in reporting practice.

It is however noted that nearly one-fifth of the patients reported the previously experienced ADRs. Ignorance of the importance of ADR reporting as well as unserious nature of the ADRs were topmost of the suggested reasons for non-report of experienced ADRs. Patients within the age of 18–40 years were significantly better in reporting experienced ADRs compared to those above 40 years. This perhaps suggests the need to further geared efforts towards continuous public education and enlightenment campaigns on the importance of prompt and timely report of experienced ADRs to healthcare professionals or used the SMS alert short code to report the experienced ADRs to the National Pharmacovigilance Centre.

Approximately one-quarter of the patients had experienced one form of ADR or the other, topmost on the list of measures taken by patients who experienced an ADR includes informing a healthcare professional, as well as stoppage of the drug(s). These actions taken by the patients may be considered as the first necessary steps whenever one experiences an ADR, before the proper investigation(s) to establish the link between the reaction and the suspect drug. Interestingly, more than half of the patients wish to report ADRs directly to a healthcare professional, while close to one-third prefer phone call or text message to a designated number for ADR reporting. This perhaps underscores the need to explore combination of approach including social media platform in ensuring sensitization and dissemination of information about early detection and reporting of ADRs.

Despite the useful information from this study, the following limitations are necessary for consideration. First, the cross-sectional nature of the study with only a snapshot of participants in the selected PHCs may lead to the possibility of selection bias especially among the patients. Also, the self-reported nature of the study may be associated with some inherent limitations such as participants who may either over- or under-report the information provided, while the possibility of recall bias may not be totally excluded. Nevertheless, self-report measure especially using a non-judgmental or non-threatening questioning approach has been described as a reliable tool to elicit a somewhat accurate information from the respondents, as it may make respondents to feel more comfortable in telling the truth [40, 41]. Another limitation of our study may be related to the relatively small number of PHCs covered in the selected LGAs which may affect the widespread generalisation of study findings to the entire PHCs in the region, though the sample size used for our study is representative of the health workers and patients in the studied PHCs. Moreover, the choice of using the model PHC in each LGA which is the prototype, as well as the design of the item-statements in the questionnaire to cover fundamental aspects of ADRs/pharmacovigilance concept may partly allow for a comprehensive exploration of health workers and patients on the subject area, hence, a useful strength for our study. Nevertheless, future study may need to put into consideration the identified gaps in order to ensure a far-reaching conclusion.

## Conclusions

It can be concluded that health workers in the selected PHCs were largely aware of pharmacovigilance but show low level of knowledge about ADRs and pharmacovigilance concept, with moderately positive attitude towards ADR reporting. Patients on the other hand demonstrate low level of awareness of pharmacovigilance and ADR reporting, with less than one-fifth who reported the previously experienced ADRs. This perhaps underscores a need for regular mandatory education and training on ADRs/pharmacovigilance concept among the PHC health workers, while continuous public enlightenment and awareness campaigns on spontaneous reporting of ADRs is advocated in order to enhance reporting rate.

## Supplementary information

**Additional file 1.** Questionnaires for the study.

## Data Availability

The datasets used and/or analysed during the current study are available from the corresponding author on reasonable request.

## Abbreviations

ADR: Adverse drug reaction
CHEW: Community health extension worker
CHO: Community health officer
EC: Ethics committee
FMOH: Federal Ministry of Health
FST: Fischer’s exact test
LG: Local government area
NAFDAC: National Agency for Food and Drug, Administration and Control
NPC: National Pharmacovigilance Centre
PCN: Pharmacists Council of Nigeria
PHC: Primary health care
PV: Pharmacovigilance
SMS: Short message service
SPSS: Statistical package for social sciences
UI: University of Ibadan
WHO: World Health Organisation
