# Supplementary material for: Awareness, knowledge, attitude and practice of adverse drug reaction reporting among health workers and patients in selected primary healthcare centres in Ibadan, southwestern Nigeria
Source: BMC Health Serv Res. 2019 Dec 3;19:926. doi: 10.1186/s12913-019-4775-9 (PMC6889459; doi:10.1186/s12913-019-4775-9)
Supplement: Supplementary file 1 — Additional file 1. Questionnaires for the study. [file 12913_2019_4775_MOESM1_ESM.doc]

**QUESTIONNAIRE ON AWARENESS, KNOWLEDGE, ATTITUDE, AND PRACTICES OF ADVERSE DRUG REACTION REPORTING AMONG PRIMARY HEALTHCARE WORKERS**

Dear Sir/Ma,

This questionnaire seeks to assess awareness, knowledge, attitude and practices of adverse drug reporting among healthcare workers in selected health facilities in Ibadan. Kindly help to respond to the questionnaire with utmost sincerity. Your response will be anonymous and confidentiality is guaranteed.

Thank you.

**SECTION A- DEMOGRAPHIC INFORMATION**

1. Sex: Female( ) Male( )
2. Age:
3. Marital status: Single( ) Married( ) Other( )
4. Profession: Nurse( ) Community Health Officer ( ) Community Health Extension Worker ( ) Junior Community Health Extension Worker( ) Senior Community Health Extension Worker ( ) Health Assistant( ) Other(please specify)
5. Level of Education: Primary leaving certificate( ) SSCE/GCE( ) OND/HND( ) B.Sc ( ) M.Sc( ) School of Health ( ) Others(please specify)
6. Work experience at the present health care center :

**SECTION B: ASSESSMENT OF GENERAL KNOWLEDGE AND AWARENESS OF PHARMACOVIGILANCE/ADVERSE DRUG REACTION**

**INSTRUCTION**: Please tick or circle the appropriate responses as it applies to you. Please note **that you can select more than one option**.

1. Have you heard about pharmacovigilance? Yes ( ) No ( )
2. If YES, how did you hear about pharmacovigilance?

Advertisement ( ) From other professionals ( ) Through trainings and seminars ( )

Others (please specify)

3.What is your understanding of pharmacovigilance?

The science and activities involved in reporting adverse drug reactions ( )

A practice focused on medication and patient safety ( )

The detection, assessment and prevention of adverse effects and other drug-related problems ( )

The process by which adverse drug reactions are monitored in a hospital ( )

The underlisted statements relate to adverse drug reactions (ADR). Please indicate your opinion to the best of your understanding.

|  | **Statements** | **Yes** | **No** | |
| --- | --- | --- | --- | --- |
| 1. | An adverse drug reaction is a side effect that is commonly experienced when patient is using a drug |  |  | |
| 2. | An adverse drug reaction is an unintended effect of the drug during its administration |  |  | |
| 3. | An adverse drug reaction is a predicted and expected reaction to a drug |  |  | |
| 4. | An adverse drug reaction is the same as a side effect |  |  | |
| 5. | An adverse reaction can be only be experienced by a patient using orthodox medicines |  |  | |
| 6. | An adverse reaction can be experienced by a patient using herbal/traditional medicines |  |  | |
| 7. | All adverse drug reactions are known before drug gets into market for use |  |  | |
| 8. | All adverse drug reactions experienced by a patient taking a drug should be reported and documented |  |  | |
| 9. | Only intolerable reactions to a drug should be reported |  |  | |
| 10. | Adverse drug reaction may not be documented if the patient was appropriately counselled against such reaction |  |  | |
| 11. | The best method of addressing adverse drug reaction is to use or recommend another drug to counter-act the drug effect |  |  | |
| 12. | There is no need to report an adverse drug reaction that is already documented in drug literature insert. |  | |  |
| 13. | Reporting and documentation of adverse drug reactions is important |  | |  |

1. What constitutes a serious adverse drug reaction to the best of your understanding?

A reaction that will lead to hospitalization ( ) A reaction that resolves on its own ( )

A reaction that is life-threatening ( ) A reaction that requires another drug treatment ( )

Others( please specify)

2. Who do you think should report an adverse drug reaction?

Any health worker ( ) Only senior health workers ( ) Patients ( ) Others ( )

3. Have you ever come across the adverse drug reporting form? Yes( ) No( )

4. Do you have the form in your health facility? Yes ( ) No( )

5. If No, what are the challenges of obtaining the form?

6. The regulatory body responsible for monitoring adverse drug reactions in Nigeria is/are? (Please tick as appropriate)

National Agency for Food and Drugs Administration and Control ( ) Pharmacists council of Nigeria( ) World Health Organization ( ) Federal Ministry of Health ( )

7. If an adverse drug reaction were to occur in your health facility, would you be able to report it? Yes ( ) No( )

8. If yes, how and where would you report such?

9. If no, please state the challenges that prevent you from such adverse drug reactions.

**SECTION C: ASSESSMENT OF ATTITUDE OF HEALTH WORKERS TO ADVERSE DRUG REACTION REPORTING**

For the following questions please tick the degree to which you agree or disagree with the question**: SA-Strongly agree , A-Agree, U-Undecided, D-Disagree SD-Strongly disagree**

|  |  | **SA** | **A** | **U** | **D** | **SD** |
| --- | --- | --- | --- | --- | --- | --- |
| 1. | I would report all adverse drug reactions I encounter |  |  |  |  |  |
| 2. | ADR reporting is part of my responsibilities as a health care professional |  |  |  |  |  |
| 3. | Training of health care professionals can aid adverse drug reaction reporting |  |  |  |  |  |
| 4. | I would be more likely to identify and report important adverse drug reactions if I received some training on pharmacovigilance |  |  |  |  |  |
| 5. | Reporting adverse drug reactions is part of my professional obligation |  |  |  |  |  |
| 6. | Pharmacovigilance should be included in training for health workers |  |  |  |  |  |
| 7. | I would be likely to report only life-threatening/severe adverse drug reactions |  |  |  |  |  |
| 8. | I would be likely to report only previously unknown adverse reactions |  |  |  |  |  |
| 9. | I do not think that tolerable , mild adverse drug reactions should be reported |  |  |  |  |  |

**SECTION D: ASSESSMENT OF ADVERSE DRUG REACTION PRACTICES AMONG HEALTHCARE WORKERS**

1. Have you ever been trained on Pharmacovigilance? Yes( ) No( )
2. If Yes, please indicate the year you received such training and the organization(s) involved.

3. If No , would you like to be trained on pharmacovigilance? Yes ( ) No ( )

4. On a scale of 1-10, please rate your interest to undergo training on pharmacovigilance

5. Do you think there are benefits of undergoing training on pharmacovigilance? Yes ( ) No ( )

6. If yes, please indicate possible advantages/benefits you are likely to obtain from partaking in pharmacovigilance training?

7. If No, what are your reasons with respect to question 5 above?

8. Have you ever experienced a situation in which a patient complained of an adverse reaction to a drug?

Yes ( ) No ( )

9. If Yes, how frequently do you encounter adverse drug reactions in your practice at the primary health center?

10. What measures do you take in case of adverse drug reaction.

Refer to a secondary health care facility ( ) Treat symptoms with another drug ( )

Nothing, reaction resolves on its own ( ) Provide counselling to patients ( )

Others(please specify)

11. Which of the following factors hinder adverse drug reaction(ADR) reporting in your primary health care center?

Reporting of adverse drug reaction is complicated ( ) Insufficient clinical knowledge ( ) Fear of liability/response ( ) No time to report ADRs ( ) ADRs I’ve seen are not life-threatening/severe enough ( ) Unavailability of form ( ) Lack of experience in filling forms ( )

Others

**QUESTIONNAIRE ON KNOWLEDGE, AWARENESS, ATTITUDE AND PRACTICES OF ADVERSE DRUG REACTION REPORTING AMONG PATIENTS IN A PRIMARY HEALTHCARE FACILITY**

Dear Sir/Ma,

his questionnaire seeks to assess knowledge, awareness, attitude, and practices of adverse drug reactions among patients attending selected primary health facilities in Ibadan. Kindly help to respond to the questionnaire with utmost sincerity. Your response will be anonymous and confidentiality is guaranteed.

Thank you.

**SECTION A- DEMOGRAPHIC INFORMATION**

1. SEX: Female( ) Male( )
2. AGE:
3. MARITAL STATUS: Single( ) Married( ) Other( )
4. OCCUPATION: Civil servant( ) Self-employed( ) Unemployed( ) Others(please specify)
5. ETHNICITY: Yoruba( ) Igbo( ) Hausa( ) Other( )
6. LEVEL OF EDUCATION : None/Primary leaving certificate( ) SSCE/GCE( ) OND/HND( ) B.Sc ( ) M.Sc ( ) Others (please specify)

**SECTION B- ASSESSMENT OF PATIENTS KNOWLEDGE AND AWARENESS OF PHARMACOVIGILANCE/ADVERSE DRUG REACTIONS**

**INSTRUCTION**: Please tick or circle the appropriate responses as it applies to you. Please note **that you can select more than one option**

1. Which of the following best describes your understanding of an adverse drug reaction:

Any effect from a medication one is using ( ) Unexpected reaction after taking a drug ( ) Expected reaction after taking a medicine ( ) I don’t know ( )

1. What constitutes a serious adverse drug reaction to the best of your understanding?

A reaction that will lead to hospitalization ( ) A reaction that resolves on its own ( ) A reaction that is life-threatening ( ) A reaction that requires another drug treatment ( ) Others( please specify)

1. Have you heard of pharmacovigilance? Yes ( ) No ( )
2. If yes, how did you hear about it

Radio ( ) Television ( ) Newspaper ( ) Social media platforms ( )

Others(please specify)

1. Have you ever heard of Pharmacovigilance Rapid Alert System for Consumer Reporting(PRASCOR)? Yes( ) No( )
2. If Yes, how did you hear about it?

Through advertisement and other online sources ( ) From friends( ) From newspaper

articles ( ) Others(please specify)

1. What is the SMS short code for reporting an ADR through Pharmacovigilance Rapid Alert System for Consumer Reporting (PRASCOR)?

20543 ( ) 23056 ( ) 20453 ( ) 20356 ( ) I don’t know( )

**SECTION C- ASSESSMENT OF PATIENTS’ PERCEPTIONS AND KNOWELEDGE OF ADVERSE DRUG REACTION REPORTING**

**INSTRUCTION**: Please tick or circle the appropriate responses as it applies to you. Please note **that you can select more than one option**

1. If you ever experience a non-serious adverse drug reaction, would you report it?

Yes ( ) No ( )

1. Why do you think patients do not report adverse drug reactions

They may not be sure if adverse effects are related to the medications being used ( )

Adverse effects may not be very serious ( )

They do not know the importance of reporting adverse drug reactions ( )

They do not know how to report such reactions ( )

Others (please specify)

1. Have you ever reported an adverse drug reaction you experienced with any drug to a health care professional?

a) Yes b)No

1. If Yes, could you briefly describe the reaction reported to the healthcare professional?
2. If No, why didn’t you report such reaction?
3. Have you ever asked the health care professional about possible adverse drug reactions you may experience from a medication? Yes ( ) No( )
4. Which of the following sources do you obtain information about adverse drug reactions?

Pharmacist ( ) Physician ( ) Drug leaflet( ) Internet ( )

1. Do you think educating patients on adverse drug reaction reporting and its importance is important? Yes ( ) No( )
2. Through which of the following means would you prefer to report adverse drug reactions you may experience

Through phone by calling or text message ( ) By filling a form( ) By filling an online form ( ) By using an online application that is designed for ADR reporting ( ) By reporting directly to health care professional ( )

**SECTION C: ASSESSMENT OF ADVERSE DRUG REACTION REPORTING PRACTICES AMONG PATIENTS**

1. Have you ever experienced an adverse reaction to any medication you took? Yes( ) No( )

1. If Yes, what was the action you took in such situation?

Did nothing because it resolved on its own ( ) Did nothing because it was tolerable( ) Stopped the drug ( ) Switched to herbal/traditional medicines ( ) Told a health care professional ( ) Others(pls specify)……………………….

1. Can you remember the particular drug you took that gave you the reaction?

Yes( ) No( )

1. Could you give a brief description of the adverse drug reaction you experienced?
